# Prehabilitation in the lung cancer pathway: a scoping review

**DOI:** 10.1186/s12885-023-11254-x

**Published:** 2023-08-11

**Authors:** Kelly Wade-Mcbane, Alex King, Catherine Urch, Julian Jeyasingh-Jacob, Andrew Milne, Clair Le Boutillier

**Affiliations:** 1 Department of Nutrition and Dietetics, Imperial College Healthcare NHS Trust, London, UK; 2 Department of Surgery and Cancer, Imperial College London, London, UK; 3 Department of Surgery, Cardiovascular and Cancer, Imperial College Healthcare NHS Trust, London, UK; 4 Medical Library, Hammersmith Campus, Imperial College London, London, UK; 5 Division of Methodologies, Florence Nightingale Faculty of Nursing, Midwifery and Palliative Care, King’s College London, London, UK; 6 THIS Institute (The Healthcare Improvement Studies Institute), University of Cambridge, Cambridge, UK

**Keywords:** Prehabilitation, Exercise, Nutrition, Wellbeing, Surgery, Oncological treatment

## Abstract

**Background:**

Lung cancer is the third most common type of cancer in the UK. Treatment outcomes are poor and UK deaths from lung cancer are higher than any other cancer. Prehabilitation has shown to be an important means of preparing patients both physically and psychologically for cancer treatment. However, little is understood about the context and mechanisms of prehabilitation that can impact physiological and psychological wellbeing.

Our aim was to review and summarise primary research on prehabilitation in the lung cancer pathway using a realist approach.

**Methods:**

A scoping review of empirical primary research was conducted. Five online medical databases from 2016 – February 2023 were searched. All articles reporting on prehabilitation in lung cancer were included in the review. For this review, prehabilitation was defined as either a uni-modal or multi-modal intervention including exercise, nutrition and/or psychosocial support within a home, community or hospital based setting. A realist framework of context, mechanism and outcome was used to assist with the interpretation of findings.

**Results:**

In total, 31 studies were included in the review, of which, three were published study protocols. Over 95% of studies featured an exercise component as part of a prehabilitation programme. Twenty-six of the studies had a surgical focus. Only two studies reported using theory to underpin the design of this complex intervention. There was large heterogeneity across all studies as well as a lack of clinical trials to provide definitive evidence on the programme design, setting, type of intervention, patient criteria, delivery, duration and outcome measures used.

**Conclusion:**

A standardised prehabilitation programme for lung cancer patients does not yet exist. Future lung cancer prehabilitation programmes should take into account patient led values, needs, goals, support structures and beliefs, as these factors can affect the delivery and engagement of interventions. Future research should consider using a conceptual framework to conceptualise the living with and beyond cancer experience to help shape and inform personalised prehabilitation services.

## Background

Lung cancer is the third most common type of cancer in the UK [1]. Treatment outcomes are poor and UK deaths from lung cancer are higher than any other cancer [2]. Cancer incidence and mortality projections within the UK predict that although mortality rates are likely to increase over the next 10 years, there will also be more people living with and beyond cancer [3].

Prehabilitation has shown to be an important means of preparing patients both physically and psychologically for cancer treatment by mitigating deconditioning associated with cancer treatments between the time of cancer diagnosis and the beginning of acute treatment’ [4, 5].

Prehabilitation within cancer surgery has shown to reduce morbidity and improve health outcomes. For example, an improvement in functional capacity [6–14] and health reported quality of life (HRQOL) [9, 12, 15, 16] as well as a reduction in post-operative complications [9, 15] and length of hospital stay [9, 13, 15, 17].

Few prehabilitation pathways exist for people who do not have surgery, despite 50–60% of people with cancer in the UK being treated with primary, neo-adjuvant or palliative chemotherapy and/or radiotherapy treatment [18]. Along with the rise in the use of targeted agents and immunotherapy, there is potential to optimise quality of life within the lung cancer population. However, little is understood about the context and mechanisms of prehabilitation that can impact physiological and psychological wellbeing. 

Prehabilitation is a complex intervention and it is widely understood that the success of a complex intervention depends on the theory underpinning its design [19], which helps to explain the mechanisms underlying an individual’s behaviour, based on what works for them and their circumstances [20–23]. Lung cancer treatment regimes can be prolonged and people may experience a range of toxicities, which could limit their ability to engage in prehabilitation interventions. Prehabilitation programmes should therefore be tailored to the individual to optimise symptom control, treatment tolerance and independence [24, 25].

The aim of this scoping review was to review and summarise primary research on prehabilitation in the lung cancer pathway using a realist approach. Realist approaches focus on the contexts and mechanisms that lead to particular outcomes. This approach enables a detailed exploration of factors likely to influence the success of a complex intervention, such as prehabilitation, thereby helping explain how and why interventions may or may not work [26, 27].

## Methods

Scoping reviews are particularly relevant to examine the extent, range and nature of evidence on a certain topic and to identify concepts, theories and knowledge gaps from a heterogeneous body of research [28].

The PRISMA extension for scoping reviews was used for the conduct and reporting of this scoping review [28]. This enabled an examination of the extent, range and nature of the evidence on prehabilitation and lung cancer.

Following the Joanna Briggs Institute (JBI) framework [29], this scoping review addressed the following: 1. Define the review questions 2. Determine the inclusion criteria 3. Search strategy 4. Evidence screening and selection 5. Data extraction 6. Data analysis 7. Presentation of the results.

### 1. Define the review questions.

Prehabilitation is a complex intervention and it is important to understand what has worked or is perceived to work based on measured or predicted outcomes within the lung cancer pathway. Pre-surgical prehabilitation is often a linear process from baseline to a defined, one-off target (surgery). However, this is not the case for patients receiving oncological treatment where prehabilitation may be delivered immediately prior to, during ± after each treatment session or cycle. Our research questions were:

A) How does the literature within the field of lung cancer describe the structure of prehabilitation?
B) How does the literature within the field of lung cancer describe the personalisation of prehabilitation interventions?
C) What are the actual outcomes for lung cancer patients participating in a prehabilitation programme?

### 2. Determine the inclusion criteria.

All studies included in this review had to involve lung cancer patients who received a form of prehabilitation within a home, community or hospital based setting. For this review, prehabilitation was defined as either a uni-modal or multi-modal intervention or programme including either exercise, nutrition and/or psychological wellbeing. All study designs were included in this scoping review providing that they met the inclusion criteria as outlined in Table 1. Protocols for ongoing or upcoming lung cancer prehabilitation studies were included in the review, as the authors felt these provided key insights into the delivery and proposed outcomes for prehabilitation within this field. All articles available in English were included.

**Table 1:** Eligibility criteria

	Inclusion criteria	Exclusion criteria
Population	Adults aged ≥ 18 years old with a diagnosis of lung cancer	Studies addressing other tumour sites
Intervention	Unimodal or multimodal* prehabilitation interventions prior to lung cancer treatment	Not applicable
Comparator	Usual care or another type of intervention	Not applicable
Outcome	The intended and unintended outcomes for lung cancer patients participating in a prehabilitation programme The effectiveness of prehabilitation programmes	Not applicable
Study design	Quantitative studies Qualitative studies Mixed method studies If relevant systematic reviews identified, primary papers will be included Study protocols for ongoing or upcoming prehabilitation studies specific to lung cancer Published from 2016 up to and including 03 February 2023	Commentaries Opinion articles Book reviews Conference abstracts Social media posts Blogs Podcasts
Language	Written in the English language	Articles published in a language other than English due to limited translation resources

*Multi-modal: delivery of two or more non-pharmacological interventions (for example exercise, nutrition and/or psychological wellbeing)

### 3. Search strategy.

The literature search was undertaken by a research librarian using pre-defined search terms between the period of 2016 and 03 February 2023. This time period was chosen due to a rapid emergence of the use of prehabilitation within cancer care to improve health outcomes and reduce healthcare costs since the publication of a key research paper by Silver in 2015 [30]. This was followed by the Macmillan prehabilitation evidence and insight review [31] and subsequent publication of the Macmillan prehabilitation guidance [32]. A total of five databases were searched incorporating medical, nursing, allied health and psychological literature relevant to prehabilitation and lung cancer: Cumulative Index to Nursing and Allied Health Literature (CINAHL), Embase, Emcare, Medical Literature Analysis and Retrieval System Online (MEDLINE) and Psychological Information Database (PsycINFO). The major search terms ‘lung’, ‘cancer’ and ‘prehabilitation’ were used.

### 4. Evidence screening and selection.

All duplications were removed using the Zotero deduplication function. All retrieved abstracts for possible inclusion were independently screened by the first and last author. There was a consensus between both authors and thus, a third reviewer was not required.

### 5&6. Data extraction and analysis.

All articles reviewed for inclusion were obtained in full text. The JBI reviewers manual for evidence synthesis was used to create a synthesis matrix for data extraction [33]. Data extraction included: study title, year of publication, country, study design, sample size, type of participants, study aim, type of prehabilitation intervention used, key findings, strengths and limitations.

## Results

**Fig. 1:**
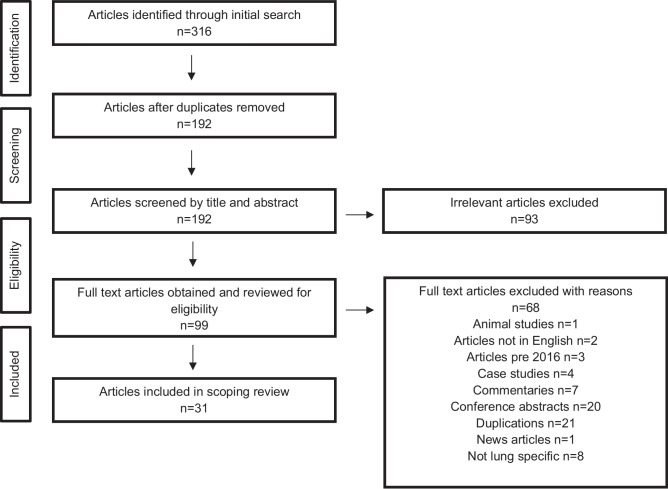
PRISMA flow diagram of the scoping review process

A total of 31 articles were included in this scoping review; see Fig. 1. In some studies, pulmonary rehabilitation was described as prehabilitation. After discussion with authors conducting the review, it was agreed that pulmonary rehabilitation is a separate intervention, acknowledging that it may complement prehabilitation in the long-term. Therefore, studies that focused on pulmonary rehabilitation were excluded from this review.

The results of this review are presented in a narrative form in Tables 2 and 3.

**Table 2:** Summary of all studies meeting the eligibility criteria and subsequently used in this present scoping review

Title and year	Country of origin	Aims and purpose	Study population / sample size		Study design/type		Key findings related to the scoping review objectives	Strengths/limitations
Prehabilitation sessions can be provided more frequently in a shortened regimen with similar or better efficacy in people with non-small cell lung cancer: A randomised trial 2022 [34]	France	To evaluate the relative effect of condensing 15 prehabilitation sessions into a 3-week regimen compared with a 5-week regimen	*N* = 36 (18 in the intervention arm and 18 in the control arm) patients with Non-Small Cell Lung Cancer (NSCLC) and moderate - high risk of postoperative complications		Randomised controlled trial		Condensing prehabilitation sessions led to similar or better improvement in cardiorespiratory fitness and did not decrease adherence or increase adverse events	Strengths: Concealed allocation, blinded assessment of the primary outcome and intention-to-treat analysis Limitations: External validity is limited with regards to inpatient or homebased programmes
Multimodal prehabilitation for lung cancer surgery: A randomised controlled trial 2021 [35]	Canada	To investigate the effect of a personalised and structured multimodal intervention on postoperative functional recovery	*N* = 95 (52 in the prehab arm and 43 in the rehab arm) lung cancer patients scheduled for NSCLC surgical resection		Randomised controlled trial		No difference in functional capacity during the perioperative period between the 2 groups Eight weeks after surgery, both groups returned to baseline functional capacity	Limitations: A four-week prehabilitation intervention may not be highly translatable to patients with short durations between diagnosis and surgery. Patients who did not speak English or French were excluded
Feasibility of a novel mixed-nutrient supplement in a multimodal prehabilitation intervention for lung cancer patients awaiting surgery: A randomised controlled pilot trial 2021 [36]	Canada	To investigate the feasibility of delivering a novel four-week multimodal prehabilitation intervention and its effects on preoperative functional capacity and Health-Related Quality Of Life (HRQOL) compared to standard hospital care	*N *= 34 (24 in the intervention arm and 10 in the control arm) patients with stage I, II or IIa lung cancer awaiting elective thoracotomy		Randomised controlled trial		A multimodal prehabilitation intervention for lung cancer patients awaiting surgery is feasible as demonstrated by 84.1% adherence to the prescribed intensity of the supervised exercise program, 88.2% adherence to the self-reported home-based exercise program and 93.2% adherence to the nutritional supplement No statistical differences in the six minute walk test or HRQOL between groups	Strengths: One of the first studies to include a specifically tailored preoperative nutritional intervention beyond dietary advice and/or whey protein supplementation in lung cancer patients undergoing surgery. Flexibility of the exercise intervention Limitations: Nature of the study design (an open-label trial) could have led to a possible placebo effect in the prehabilitation group and self-supplementation in the control group. Due to the behavioural nature of the intervention, control patients may have sought out similar exercises on their own, as evidenced by the significant increase in self-reported moderate -vigorous intensity physical activity. High proportion of missing data at the preoperative visit, due to the COVID-19 pandemic. No data on adherence to the anxiety-reducing component of the intervention
Preoperative exercise to improve fitness in patients undergoing complex surgery for cancer of the lung or oesophagus (PREHIIT): Protocol for a randomised controlled Trial 2020 [37]	Ireland	To examine the influence of exercise prehabilitation on physiological outcomes and postoperative recovery and through evaluation of health economics, the impact of the programme on hospital costs	*N* = 78 (39 in the intervention arm and 39 in the control arm) patients scheduled for either an oesophagectomy or major lung resection		Randomised controlled trial (Protocol)		Planned outcomes: Primary outcomes: Cardiorespiratory fitness. Secondary outcomes: Post-operative morbidity, pulmonary and physical performance and quality of life	Strengths: A randomised controlled trial. An examination of the economic implications of the exercise prehabilitation programme will be done as part of the outcomes Limitations: Absence of nutritional screening despite nutritional adequacy being examined. Inclusion of both lung and oesophageal cancer patients
Effects of an exercise intervention for patients with advanced inoperable lung cancer undergoing chemotherapy: A randomised clinical trial 2020 [38]	Denmark	To evaluate the effect of a physical exercise program comprising 12 weeks of supervised, structured aerobic, strength, and relaxation training twice weekly for patients with advanced inoperable lung cancer	*N* = 218 (110 in the intervention arm and 108 in the control arm) patients with stage IIIb-IV NSCLC		Randomised controlled trial		No significant difference between the intervention and control group in VO2 peak. Statistically significant difference in strength; leg press (*p* = 0.01), leg extension (*p* = < 0.01), chest press (*p* = < 0.01) and lateral pull down (*p* = 0.04) and in social well-being (*p* = 0.04), anxiety (*p* = 0.02) and depression (*p* = 0.01) between the intervention and control group	Limitations: 36.6% drop out rate. Adherence to the intervention was only 44%. Patients unable to attend the hospital for the exercise program were excluded
Cost-effectiveness of a technology supported multimodal prehabilitation program in moderate-to-high risk patients undergoing lung cancer resection: Randomised controlled trial protocol 2020 [39]	Spain	To evaluate the cost-effectiveness of a multimodal prehabilitation program supported by information and communication technologies	*N* = 158 (79 in the intervention arm and 79 in the control arm)		Randomised controlled trial (Protocol)		Planned outcomes: (i) Patient and professional experience, (ii) Population health-based outcomes (e.g. hospital length of stay, number and severity of postoperative complications, peak oxygen uptake and levels of systemic inflammation) and (iii) Healthcare costs	Strengths: One of the first studies to look at cost-effectiveness and factors which could modulate service adoption Limitations: Patients without access to a smartphone will be excluded
Preoperative respiratory muscle endurance training improves ventilatory capacity and prevents pulmonary postoperative complications after lung surgery 2020 [40]	France	To evaluate preoperative Respiratory Muscle Endurance Training (RMET) on respiratory muscle capacity and postoperative complications		*N* = 26 patients (14 in the intervention arm and 12 in the control arm) undergoing lung resection for NSCLC	Randomised controlled trial		Statistically significant improvement in respiratory muscle endurance (*p* = 0.001) and reduction in pulmonary postoperative complications (*p* = 0.037) in the intervention group compared to the control group	Strengths: One of the first studies to demonstrate the benefits of prehabilitation via RMET Limitations: Small sample size. Possible selection bias – highly motivated group of participants
Impact of one-week preoperative physical training on clinical outcomes of surgical lung cancer patients with limited lung function: A randomised trial 2019 [41]	China	To investigate the influence of preoperative physical training combining aerobic and breathing exercises on surgical lung cancer patients with impaired lung function		*N* = 68 (34 in the intervention arm and 34 in the control arm) NSCLC patients	Randomised controlled trial		Preoperative physical training (aerobic and breathing exercises) can improve exercise capacity, as evidenced by a statistically significant increase in distance covered by the 6 min walk test (6MWD) (*p* = 0.004), reduction in post-operative pulmonary complications (*p* = 0.022), shorter length of stay (*p* = < 0.001) and lower in-hospital costs (*p* = 0.016). No statistically significant differences in lung function or quality of life, except for the emotional function of the EORTC QLQ-C30 (*p* = 0.001) between groups	Strengths: Inclusion of a cost-effectiveness analysis of the intervention Limitations: Generalisability of findings is limited as it’s a small single centre study with the inclusion of non NSCLC patients
Pre-operative high intensity interval training is effective and safe in deconditioned patients with lung cancer: A randomised clinical trial 2019 [42]	Switzerland	To examine the effect of prehabilitation in patients diagnosed with lung cancer		*N* = 151 (74 in the intervention arm and 77 in the control arm) patients with NSCLC stage IIIa or less	Randomised controlled trial		Short-term high intensity interval training is feasible in deconditioned patients as evidenced by 87% adherence. It increases cardio-respiratory fitness and walking capacity as evidenced by a statistically significant increase in high-intensity interval training power (*p* = < 0.001), heart rate (*p* = < 0.01), aerobic capacity (*p* = 0.004) and 6MWD (*p *= < 0.001) in the intervention group compared to usual care	Strengths: Demonstrated benefit of exercise prehabilitation in a short time period (8 training sessions over 3 weeks). High adherence. Limitations: Absence of nutrition and psychological components of prehabilitation
Two week multimodal prehabilitation program improves perioperative functional capability in patients undergoing thoracoscopic lobectomy for lung cancer: A randomised controlled trial 2019 [43]	China	To investigate the effects of a two week, homebased, multimodal prehabilitation program on perioperative functional capacity and other outcomes		*N* = 73 (37 in the intervention arm and 36 in the control arm) patients with stage I-III NSCLC undergoing Video Assisted Thoracoscopic Surgery (VATS) lobectomy	Randomised controlled trial		Statistically significant improvement in the 6 min walk test (6MWT) (*p* = < .001) and forced vital capacity (*p* = .0.21) in the intervention group compared to the control group (*p* = < .001) No difference in lung function, disability, psychological assessment, length of stay, postoperative complications and mortality	Strengths: Tri modal prehabilitation programme (nutrition, exercise and psychology) Limitations: Patients only included if they were having a VATS lobectomy, all other lung cancer surgeries were excluded. No information on lifestyle changes
Precision-Exercise-Prescription (PEP) in patients with lung cancer undergoing surgery: Rationale and design of the PEP study trial 2018 [44]	United States of America	To test the clinical effectiveness and feasibility of a personalised exercise intervention in patients with lung cancer undergoing surgery		*N* = 200 (100 in the intervention arm and 100 in the control arm) patients with primary lung cancer stage I, II or IIIa or secondary lung cancer undergoing surgery	Randomised controlled trial (protocol)		Planned outcomes: Primary outcome: Level of physical function assessed by the 6MWT at 2 months post-surgery. Secondary outcomes: Quality Of Life (QOL), fatigue, self-efficacy, length of stay, complications, readmission, pulmonary function and treatment-related costs up to 6 months post-surgery	Strengths: One of the first RCT’s to examine a personalised exercise programme for patients with primary and secondary lung cancer across the continuum of care from pre surgery to post surgery. Limitations: Patients unable to follow directions and complete questionnaires and exercise diaries in English would be excluded
A comparison of the effects of medical Qigong and standard exercise therapy on symptoms and quality of life in patients with advanced cancer 2017 [45]	Canada	To compare the impact of medical Qigong and standard exercise therapy on anxiety and depression levels and QOL and to establish whether medical Qigong or standard exercise training is superior for improving functional capacity and reducing symptoms		*N* = 24 patients with stage three or four NSCLC or gastrointestinal cancer	Randomised controlled trial		No significant differences in anxiety, depression or QOL between Qigong and standard exercise therapy. However, standard exercise therapy was superior to Qigong as demonstrated by statistically significant improvements in perceived strength (*p* = 0.05) and walking distance (*p* = 0.02)	Strengths: One of the first studies to compare Qigong with another exercise intervention Limitations: Study was underpowered. Only 19 patients completed both assessments
Impact of physical activity on fatigue and quality of life in people with advanced lung cancer: A randomised controlled trial 2017 [46]	Australia	To assess whether a 2-month physical activity intervention improves fatigue and QOL		*N* = 112 (56 in the intervention arm and 55 in the control arm) patients with stage III/IV NSCLC or Small Cell Lung Cancer (SCLC)	Randomised controlled trial		No significant differences in fatigue, QOL, symptoms, physical or functional status or survival between the groups	Strengths: Physical activity intervention adherence was good (69%), demonstrating that advanced lung cancer are able to participate in structured physical activity programmes Limitations: Selection bias – motivated group
Preoperative exercise training prevents functional decline after lung resection surgery: A randomised, single-blind controlled trial 2017 [47]	Spain	To investigate the effects of a preoperative exercise programme in patients with lung cancer undergoing VATS		*N* = 22 (10 in the intervention arm and 12 in the control arm) patients with suspected or confirmed diagnosis of NSCLC	Randomised controlled trial		Statistically significant improvement in exercise tolerance (*p* = 0.0001), the physical summary component of the Short Form (SF) 36 survey (*p* = 0.008) and muscle strength (*p* = < 0.01) in the intervention arm compared to the control group. There were no significant differences between groups after surgery. However, 3 months post-surgery, there were statistically significant differences in mean change of exercise capacity (*p* = 0.005), physical summary component (*p* = 0.001) of the SF-36 and upper (*p* = 0.045) and lower body strength (*p* = 0.002)	Strengths: Demonstrated that a preoperative exercise based programme can improve exercise capacity, muscle strength and the physical component of health related quality of life in patients with lung cancer Limitations: Small study with a high dropout rate – of the 20 patients randomised to the prehabilitation group, only 50% completed the study
Short-term preoperative exercise therapy does not improve long-term outcome after lung cancer surgery: A randomised controlled study 2017 [48]	Switzerland	To evaluate the impact of adding rehabilitation with High Intensity Interval Training (HIIT) before lung cancer surgery to enhance cardio-respiratory fitness and improve long-term postoperative outcome		*N* = 151 (74 in the intervention arm and 77 in the control arm) patients with suspected or confirmed stage IIIa or less NSCLC	Randomised controlled trial		A HIIT program before lung cancer resection did not improve clinical and functional outcomes at 1 year after surgery as evidenced by no statistical significant difference in survival, pulmonary function or cardiorespiratory difference between the 2 groups	Strengths: One of the first studies to examine the impact of prehabilitation on long-term outcomes after resection Limitations: A third of patients did not complete the pulmonary function and cardiopulmonary exercise tests for unknown reasons
Short-term preoperative high-intensity interval training in patients awaiting lung cancer surgery: A randomised controlled trial 2017 [49]	Switzerland	To evaluate and compare short-term preoperative HIIT to usual care by assessing cardio-respiratory fitness parameters and the incidence of postoperative complications		*N* = 151 (74 in the intervention arm and 77 in the control arm) patients undergoing NSCLC resection	Randomised controlled trial		Preoperative HIIT resulted in statistically significant improvements in peak oxygen consumption (*p* = 0.003) and 6MWD (*p* < 0.001) There were no statistically significant differences in complication rates between groups	Strengths: Demonstrated the safety and effectiveness of a short-term exercise training program in improving aerobic performance Limitations: Study was underpowered. 25% did not experience any benefit in aerobic fitness despite attending the prescribed training sessions
Is preoperative protein-rich nutrition effective on postoperative outcome in non-small cell lung cancer surgery? A prospective randomised study 2016 [50]	Turkey	To investigate the benefit of preoperative nutritional support for NSCLC patients who underwent anatomic resection		*N* = 58 (31 in the intervention arm and 27 in the control arm) patients with NSCLC	Randomised controlled trial		Preoperative nutrition provided a statistically significant reduction in complications (*p* = 0.049) and chest tube removal time (*p* = 0.019) in patients who were in the intervention arm compared to the control group There was also a statistically significant reduction of 25% in postoperative albumin levels in patients in the control arm compared to only a 14% reduction in those in the intervention arm (*p* < 0.001)	Strengths: One of the first studies examining the effect of nutritional prehabilitation in patients with NSCLC Limitations: Disproportionate number of males versus females; 54 versus 4 respectively. Excluded malnourished patients and patients with a low Body Mass Index (BMI). Changes in albumin could be multifactorial
Effect of prehabilitation on ventilatory efficiency in non–small cell lung cancer patients: A cohort study 2019 [51]	France	To assess the effect of prehabilitation on the minute ventilation / carbon dioxide production slope (VE/VC02) and its relationship with VO2peak		*N* = 50 patients with NSCLC	Cohort study	No statistically significant change in VE/VCO2 slope (*p* = 0.09), length of hospital stay (*p* = 0.55) and post-operative complications (*p* = 0.50) from baseline to post prehabilitation. However, there was a statistically significant increase in VO2peak (*p* = 0.01) and cardiorespiratory parameters (*p* = 0.01) from baseline to post prehabilitation		Strengths: One of the first studies to evaluate the effect of prehabilitation before lung resection for NSCLC on VE/VCO2 slope Limitations: Retrospective study design. Exercise intensity was not accounted for
Impact of prehabilitation on morbidity and mortality after pulmonary lobectomy by minimally invasive surgery: A cohort study 2018 [52]	France	To determine whether participation in a prehabilitation program would improve outcomes after surgery and lower morbidity according to the Clavien-Dindo classification		*N* = 38 (19 in the intervention arm and 19 in the control arm) patients with NSLC stage IIIA or less who had pulmonary lobectomy by minimally invasive surgery	Cohort study	Statistically significant difference in favour of the prehabilitation group with a Clavien-Dindo grade of ≤ 2 (*p* = 0.02) and fewer postoperative complications (*p* = 0.03) No significant difference between the two groups in length of stay and severity of complications		Strengths: One of the first studies to focus on prehabilitation and the severity of post-operative complications using the Clavien-Dindo classification Limitations: Small sample size. Post-operative complications were only recorded up to 30 days post-surgery. Patients unable to attend the hospital for the exercise program were excluded
Application and practice of trimodal prehabilitation model in preoperative management of patients with lung cancer undergoing video-assisted thoracoscopic surgery 2023 [53]	China	To analyse the application of trimodal prehabilitation model in preoperative management of patients with lung cancer undergoing VATS		*N* = 148 (74 in the intervention arm and 74 in the control arm) stage I-II NSCLC patients due to undergo VATS	Prospective study	Statistically significant improvement in the 6MWT and activity levels (p values not provided) and a reduction in the Hospital Anxiety Depression Scale (HADS) and post-operative complications (*p* values not provided) between groups post intervention		Strengths: Tri modal prehabilitation programme (nutrition, exercise and psychology) Limitations: Results to be interpreted with caution owing to the absence of *P* values
Pre-treatment optimisation with pulmonary rehabilitation in lung cancer: Making the inoperable patients operable 2021 [54]	United Kingdom	To determine whether pre-operative prehabilitation, by improving clinical parameters, (i) makes patients suitable for surgery who were considered inoperable and (ii) thereby allows them to safely receive curative surgery with reduced morbidity and mortality		*N* = 216 lung cancer patients	Prospective study	Clinically and statistically significant improvement in dyspnoea scores (*p* = 0.00002) performance status (*p* = 0.003) level of activity (*p* = < 0.00001) and frailty (*p* = 0.00058) from baseline to post prehabilitation intervention 42.8% underwent surgery following prehabilitation		Strengths: Prehabilitation intervention is as short as 2 weeks, which is more translatable to patients with short durations between diagnosis and surgery. Prospective study so all data is collected in real time Limitations: Lack of a control group. No information on nutrition
Neoadjuvant prehabilitation therapy for locally advanced non–small-cell lung cancer: Optimizing outcomes throughout the trajectory of care 2022 [55]	Canada	To assess whether neoadjuvant prehabilitation helps to optimise outcomes		*N* = 141 (20 in the intervention arm and 121 in the control arm) lung cancer patients who underwent neoadjuvant treatment followed by surgery	Retrospective study	Statistically significant improvement in the 6WMT (*p* = .1), self-reported functional status (*p* = .03) and HADS (*p* = .005) in the intervention group compared to the control group		Strengths: Tri modal prehabilitation programme (nutrition, exercise and psychology). Use of validated tools for assessment of nutrition, exercise and psychological wellbeing Limitations: Significant difference in the number of patients between both arms
Malnourished lung cancer patients have poor baseline functional capacity but show greatest improvements with multimodal prehabilitation 2021 [56]	Canada	To characterise the presence of malnutrition, examine the association between malnutrition and baseline functional capacity and the extent to which patients benefit from preoperative multimodal prehabilitation		*N* = 162 (number in the prehabilitation group vs control not specified) lung cancer patients undergoing lung cancer resection	Retrospective study	High nutrition risk patients had significantly lower baseline functional capacity compared with those who were low risk (*p* = 0.022), but experienced significant improvements in preoperative functional capacity upon receiving multimodal prehabilitation (*p* = 0.01)		Strengths: One of the first prehabilitation studies to look at the association between patients categorised as being high nutritional risk and functional capacity Limitations: High proportion of missing data (30%). Change in nutritional status was not measured, as the assessment tool was not repeated at follow up visits. Study was not powered
Feasibility and outcomes of a real-world regional lung cancer prehabilitation programme in the UK 2022 [57]	United Kingdom	To evaluate the feasibility, uptake and outcomes of the Prehab 4 Cancer service delivery model during the 11 months before COVID-19 restrictions		*N* = 377 lung cancer patients with a treatment recommendation of surgical resection	Feasibility study	The programme was feasible at scale with high uptake and had a positive impact on preoperative physiological and subjective functional assessments, providing a framework for wider implementation		Strengths: Multi- disciplinary team approach. Triage based on the principles of NHS England’s personalised care model Limitations: Risk of selection bias owing to no control group. Incomplete end of prehabilitation assessment data. Only 120 patients completed the programme; 1 in 5 opted not to participate in the programme and the reasons for this are poorly understood
Feasibility of setting up a pre-operative optimisation ‘prehab’ service for lung cancer surgery in the UK 2020 [58]	United Kingdom	To assess the feasibility of setting up a prehabilitation service for lung cancer surgery		*N* = 50 lung cancer patients due to undergo surgery	Feasibility study	A lung cancer prehabilitation programme is feasible and safe as demonstrated by a statistically significant improvement in forced expiratory volume (*p* = 0.0045), 6MWT (*p* = < 0.0001), sit to stand (*p* = 0.0011) and QOL (*p* = 0.0213) scores from baseline to post prehabilitation intervention and no adverse events		Strengths: Use of the Orsmond and Cohn framework for feasibility studies to assess feasibility
A feasibility study of an unsupervised, pre-operative exercise program for adults with lung cancer 2020 [59]	United States of America	To explore the feasibility, acceptability and perceived utility of the provision of a wearable fitness device and an exercise prescription from a surgeon		*N* = 30 patients with stage I, II or III lung cancer scheduled for surgery	Feasibility study	A wearable fitness device and exercise prescription is feasible and acceptable as evidenced by 79% completing the pre-operative study activities. 71% successfully synchronised their device during the pre-operative period. Data was transmitted from the device to the study team for an average of 70% of pre-operative days		Strengths: High engagement with the device and study assessments Limitations: Some patients voiced confusion as to how to use the device, which could limit future trials with older populations. Questions surrounding the generalisability of findings as patients who were willing to enrol on the programme may be more active than an average lung cancer patient. Patients who did not speak English were excluded
Pre-radiotherapy daily exercise training in non-small cell lung cancer: A feasibility study 2019 [60]	Denmark	To examine the feasibility of an individual, supervised, structured moderate-to-high intensity cycle ergometer exercise training immediately before radiotherapy		*N* = 15 patients with locally advance NSCLC	Feasibility study	Feasible and safe as demonstrated by 90% attendance to exercise, 88% adherence to full exercise participation and no adverse events		Strengths: First study to test whether daily individualised structured exercise is feasible and safe in patients with advance NSCLC. Ease of access for patient participation Limitations: Small sample size. Selection bias – motivated group
Patients’ and healthcare professionals’ views on a pre-and post-operative rehabilitation programme (SOLACE) for lung cancer: A qualitative study 2021 [61]	United Kingdom	To explore patients and healthcare professionals views and experiences of a pre-and post-operative rehabilitation intervention		*N* = 25 (17 patients who had early-stage lung cancer and had surgery + 8 healthcare professionals who work with lung cancer patients in pre and post-surgical care)	Qualitative study	The SOLACE service was positively perceived by patients and healthcare professionals. Patients valued the provision of tailored support/advice and peer support and reported benefits to their health and well-being Barriers to patient uptake of the classes included time constraints, motivation and access for patients who lived at a distance Virtual support may address equality of access to service for those who live at a distance from the hospital		Strengths: Provided an understanding of the value of a pre and post rehabilitation programme. The semi-structured interviews shed light on what was acceptable as well as the barriers to participation Limitations: Views of patients who did not participate in the exercise classes were not represented in the interviews. Individuals who did participate in the exercise programme were more likely to be highly motivated to participate. Not an ethnically diverse group, therefore generalisability of the findings is limited. Equality of access to the service for those who live a distance away from the hospital
Attitudes and perceptions to prehabilitation in lung cancer 2020 [62]	Australia	To determine the acceptability and perceived benefit of prehabilitation in lung cancer among thoracic surgeons		*N* = 28 thoracic surgeons	Online cross-sectional survey	91% were willing to delay surgery to optimise patients via prehabilitation The main barriers to prehabilitation were patient comorbidities and access to allied health professionals 92% believe that further research into prehabilitation in lung cancer is warranted		Strengths: One of the first studies to look at the attitudes and perceptions of prehabilitation amongst thoracic surgeons Limitations: Response rate was only 14%
Potential effectiveness of a surgeon-delivered exercise prescription and an activity tracker on pre-operative exercise adherence and aerobic capacity of lung cancer patients 2021 [63]	United States of America	To determine the level of Moderate-Vigorous Physical Activity (MVPA) and change in aerobic capacity after participation in a home-based pre-operative exercise intervention		*N* = 18 patients with stage I-III lung cancer due to undergo surgery	Proof of concept study	Mean MVPA per day: 20.4 min during the pre-operative period. On average, patients met the goal of 30 min of MVPA on 16.4% of the days during the pre-operative period No statistical significant difference found in the 6MWT between baseline and post MVPA (*p* = 0.14). 47% demonstrated a clinically significant improvement of 14 m or more, highlighting that a surgeon-delivered exercise prescription plus an activity tracker may promote clinically significant improvement in aerobic capacity and MVPA engagement		Strengths: One of the first studies to look at the level of adherence when exercise is enthusiastically prescribed by a surgeon and objectively measured Limitations: Patients who did not have access to the internet were excluded. An objective measure of pre-intervention MVPA to compare MVPA levels during the intervention was not obtained. The Garmin Vivoactive heart rate device used had not been validated for MVPA assessment among the general population or among pre-operative cancer patient populations
Prehabilitation in thoracic cancer surgery: From research to standard of care 2021 [64]	Canada	To determine whether personalised, stepped prehabilitation care is a feasible, safe, and effective implementation strategy		*N* = 81 (45 in the intervention arm and 36 in the control arm) lung cancer patients due to undergo lung cancer surgery	Quality Improvement Project	A personalised, stepped prehabilitation program targeting high-risk patients undergoing elective lung cancer surgery is feasible, safe, and effective as evidenced by a statistically significant improvement in 6MWD (*p* = 0.001), oxygen uptake (*p* = 0.004) and hospital length of stay (*p* = 0.101) after prehabilitation. There were no significant differences in the number of complications and there were no adverse events		Strengths: Tri-modal (nutrition, exercise and psychological wellbeing) prehabilitation approach Limitations: Selection bias - only patients who lived in the metropolitan area and had an expected waiting time of about four-to-five weeks before surgery were referred. Limited external validity as no predefined and universal outcome variables used or consistent time points. The intervention and control groups were not balanced

**Table 3:** Description of the prehabilitation interventions using a modified version of the TIDieR checklist

Study	What			Who provided				How	Where			When and how much						Tailoring	How well (actual/planned)
	**Components**	**Description**																	
Prehabilitation sessions can be provided more frequently in a shortened regimen with similar or better efficacy in people with non-small cell lung cancer: A randomised trial (Randomised controlled trial) [34]	• Exercise • Wellbeing Not based on a model or theory, but on the findings of a previous study which found that patients who completed ≥ 15 sessions showed more improvement in cardiorespiratory measures after the prehabilitation program than those who performed fewer sessions	Exercise: Consists of aerobic endurance training on a cycle ergometer, peripheral muscle strengthening and inspiratory muscle strengthening Wellbeing: Smoking cessation support and education on mucus clearance techniques, deep-breathing, directed and protected coughing, and postoperative mobilisation		Physiotherapist				Exercise: One to one and group sessions Wellbeing: Support and education was delivered during the first individual session and reminders and advice were provided during the following group sessions	Information not provided			Intervention arm: 5 × 90-min sessions per week for 3 weeks Control arm: 3 × 90-min sessions per week for 5 weeks						Load / intensity / resistance increased according to individual tolerance	Mean estimates of VO2peak and VE/VCO2 slope favoured the dense prehabilitation regimen and the confidence intervals indicated that the effects are as good as or better than the control regimen. However, this was not associated with a reduction in postoperative complications in this study
Multimodal prehabilitation for lung cancer surgery: A randomised controlled trial (Randomised controlled trial) [35]	• Exercise • Nutrition • Wellbeing Not based on a model or theory, but research that although exercise training has been shown to significantly improve physical function, lung cancer patients are at nutritional risk due to a reduced food intake and often experience psychological stress which could lead to a delayed recovery and mortality	Exercise: Moderate-vigorous intensity aerobic training and resistance training Nutrition: Patients screened using validated tools, advised to aim for 1.5 g/kg/d of protein and prescribed whey protein supplements if required Wellbeing: Relaxation exercises based on imagery, visualisation and deep breathing to help reduce anxiety		Exercise: Certified kinesiologist Nutrition: Registered dietitian Wellbeing: Psychology trained personnel				Exercise: Personalised exercise prescription Nutrition: All patients screened using the Patient Generated Subjective Global Assessment (PG-SGA) and the Nutritional Risk Screening tool. Daily protein calculated at 1.5 g/kg ideal body weight Wellbeing: A compact disc with relaxation exercises provided	Home-based unsupervised programme			Immediately after baseline assessment (approximately 4 weeks prior to surgery) and up to 8 weeks after surgery Exercise: 30 min of moderate-vigorous intensity aerobic training 3 days a week + resistance training (8–12 repetitions) and stretching exercises 3 days a week Nutrition: 1.5 g/kg ideal body weight of protein per day + protein supplements within 1 hour of exercise Wellbeing: 2–3 times a week						Exercise: Tailored to a patient’s preferred type of exercise and fitness level Nutrition: Advice given based on a 3 day food diary completed at the time of enrolment Wellbeing: Information not provided	No difference in the trajectory of functional capacity post-surgery No difference in median hospital length of stay, but 42% versus 16% were discharged post-operatively by day two. No discussion on the influence of nutritional optimisation and anxiety reducing strategies
Feasibility of a novel mixed-nutrient supplement in a multimodal prehabilitation intervention for lung cancer patients awaiting surgery: A randomised controlled pilot trial (Randomised controlled trial) [36]	• Exercise • Nutrition • Wellbeing Not based on a model or theory, but the hypothesis that during the preoperative period the novel multimodal prehabilitation intervention would be feasible and improve preoperative functional capacity compared to standard hospital care	Exercise: Consisted of supervised and unsupervised moderate aerobic exercise and resistance exercise Nutrition: Individualised dietary assessment to meet protein intake of > 1.2 g/kg/d and energy of 25–30 kcal/kg/d + a whey protein isolate supplement with leucine + a daily fish oil supplement with vitamin D Wellbeing: Relaxation exercises based on imagery, visualisation and deep breathing		Exercise: Kinesiologist Nutrition: Dietitian Wellbeing: Psychology trained personnel				Exercise: Personalised exercise prescription Nutrition: One to one dietary assessment based on intake, anthropometry, nutrition-impact symptoms, biochemistry and a nutrition-focused physical exam Wellbeing: One to one sessions and patients were given a compact disc with relaxation exercises to be performed at home. Adherence was assessed based on responses in a patient information booklet	Hospital based combined with unsupervised sessions at home			4 weeks prior to surgery: Supervised exercise: 1 h / week of aerobic exercise + resistance exercises: 1–2 sets of 8–15 repetitions for 30 min Unsupervised exercise: 30 min of moderate aerobic exercise + resistance exercises every second day Nutrition: Whey protein supplementation twice a day Wellbeing: 2–3 times a week						The exercise program was individualised based upon initial assessments. Dietary advice was individualised	High adherence rates to suggest feasibility for the exercise and nutritional component, but no data on the psychological component Recruitment rate was 58.6% No improvement observed in preoperative functional capacity
Preoperative exercise to improve fitness in patients undergoing complex surgery for cancer of the lung or oesophagus (PREHIIT): Protocol for a randomised controlled trial (Randomised controlled trial protocol) [37]	• Exercise • Nutrition Not based on a model or theory, but based on preliminary evidence that 12–15 sessions of HIIT significantly improves cardio-pulmonary fitness in low-fit older adults undergoing lobectomy and hepatic resection, however further evaluation in larger cohorts and in those with highest postoperative risk is required	Exercise: Supervised HIIT programme Nutrition: Tailored dietetic assessment to ensure nutritional adequacy is maintained throughout the intervention		Exercise: Physiotherapist Nutrition: Dietitian				Exercise: One to one supervised sessions on a cycle ergometer at a time convenient to the patient Nutrition: Ensuring an adequate dietary energy (25-30 kcals/kg/day) and protein intake (1.25–1.5 g/kg/day)	Hospital based			Exercise: At least 2 weeks up to 5 days a week with each session lasting 40 min Nutrition: Information not provided						Exercise and dietary intervention will be tailored to the individual	The following will be measured at diagnosis, baseline and post intervention prior to surgery: cardio-pulmonary fitness (CPET), pulmonary and physical performance (maximal inspiratory pressure, peripheral muscle strength, short physical performance battery, International Physical Activity Questionnaire) and QOL (EORTC). The following will be measured at post-operative recovery: post-operative morbidity index, post-operative morbidity (Clavien-Dindo classification), complex complications index, mortality, length of stay, self-reported functional recovery at 30 days and QOL (EORTC, EQ5DL). A sub cohort of patients will take part in a semi-structured interview to feedback on how the study has impacted their preparation for surgery. Economic evaluation will also be undertaken. Discharge destination, use of community health services and EQ5D5L scores will be collected to assess if the intervention has longer term effects beyond discharge
Effects of an exercise intervention for patients with advanced inoperable lung cancer undergoing chemotherapy: A randomised clinical trial (Randomised controlled trial) [38]	• Exercise No model or theory base reported	Physical training and relaxation comprising of strength training, aerobic training and stretching		A clinical nurse specialist or physiotherapist screened all patients prior to participation. A research physiotherapist delivered the exercise training				Supervised group training consisting of stationary cycling and strength training	Information not provided			2 × a week for 1.5 h over 12 weeks						The exercise sessions were tailored to the patient’s fitness level	Attrition rate was 37% due to death (*n* = 12), refusal to participate (*n* = 22), disease progression (*n* = 20), and absence from test (*n* = 27). All patients were undergoing concurrent systemic treatment and 67% received radiotherapy. No significant difference seen in the primary outcome; peak V02
Cost-effectiveness of a technology supported multimodal prehabilitation program in moderate-to-high risk patients undergoing lung cancer resection: Randomised controlled trial protocol (Randomised controlled trial) [39]	• Exercise • Nutrition • Wellbeing Not based on a model or theory, but the hypothesis that surgical lung cancer patients are likely to benefit from prehabilitation as they usually have a significant reduction in functional capacity from multifactorial origin	Intervention arm: Exercise: High-intensity endurance exercise, strength training and a personalised pedometer based program. Nutrition: Individualised dietary counselling to meet 1.5–2 g/kg/d of protein + a whey protein powder or casein supplement Wellbeing: Smoking cessation and cognitive behavioural therapy Control arm: patients will receive advice on physical activity, smoking cessation and alcohol intake. If deemed at risk of malnutrition, patients will receive nutritional intervention		Exercise: Physiotherapist Nutrition: Dietitian Wellbeing: Clinical health psychologist				Exercise: One to one on a cycle ergometer and use of a physical activity tracker linked to a mobile app Nutrition: Personalised dietary counselling with educational material and follow-up surveys in the mobile app Wellbeing: Group sessions and audio guides for coping strategies and exercises in the mobile app	Community based and via a mobile app			Prior to surgery. Duration not specified Exercise: 3 × week Nutrition: Information not provided Wellbeing: 1 × week						Exercise: Endurance training will be tailored to the individual according to symptoms (using the modified Borg scale) and the strength training will be adapted to an individual’s tolerance Nutrition: Individualised dietary assessment Wellbeing: Information not provided	Study outcomes to follow a quadruple aim approach. (1) Patient experience (Person Centred Coordinated Experience Questionnaire, Nijmegen Continuity Questionnaire and focus groups and structured interviews to identify facilitators and barriers to prehabilitation) (2) Population health-related outcomes (Length of stay, postoperative complications, readmissions, physical activity, wellbeing and nutrition markers) (3) Healthcare costs (4) Healthcare professionals’ perspective (Advancing Care coordination and Telehealth deployment at Scale questionnaire, focus groups and structured interviews)
Preoperative respiratory muscle endurance training improves ventilatory capacity and prevents pulmonary postoperative complications after lung surgery (Randomised controlled trial) [40]	• Exercise Not based on a model or theory, but based previous RCTs which have showed significant improvements in respiratory muscle endurance and exercise capacity in patients with Chronic Obstructive Pulmonary Disease (COPD) patients following RMET	RMET, consisting of isocapnic hyperpnoea and usual pre-operative chest therapy		A physical therapist			Using a Spirotiger® device. The RMET was supervised once a week by the same physical therapist		Hospital and home based			Pre-operatively. Patients receive 12 sessions of RMET over three weeks and are asked to complete a 30-min training session daily. RMET is performed on 2 consecutive days and rested for 1 day						RMET was tailored to each patient via use of the Spirotiger® device	86% adherence to the training programme. Minute ventilation and endurance time increased significantly after RMET. The number of post-operative complications was significantly lower in those who received RMET
Impact of one-week preoperative physical training on clinical outcomes of surgical lung cancer patients with limited lung function: A randomised trial (Randomised controlled trial) [41]	• Exercise No model or theory base reported	Consisted of physical training including breathing exercises and aerobic exercise		Nurse specialists and physical therapists				Breathing exercises: Using a volumetric incentive spirometer Aerobic exercise: Using a Nu-Step instrument	Information not provided			The intervention was provided over 1 week. Breathing exercises: 3 × day and aerobic exercise: 30 min a day						Information not provided	Significant difference in the distance covered in the 6MWT in the intervention arm compared to the control group, indicating that short-term high-intensity training regimen could improve cardiopulmonary endurance. No significant difference in lung function or quality of life (except for emotional function) between the two groups, suggesting that intense training before surgery is only effective on mental health
Pre-operative high intensity interval training is effective and safe in deconditioned patients with lung cancer: A randomised clinical trial (Randomised clinical trial) [42]	• Exercise Not based on a model or theory, but research that HIIT has shown to be feasible and safe in deconditioned patients with chronic heart disease and this could be replicated in patients awaiting primary lung resection surgery for NSCLC	HIIT exercise program	Respiratory physiotherapists					Supervised group sessions using a cycle ergometer	Information not provided			3 weeks prior to surgery; 30 min 1–3 times a week					If patients were unable to complete sessions at 100% power, the power was lowered according to the patients capacity to obtain a dyspnoea and leg fatigue of at least 5 on the Borg scale. Power was increased again if dyspnoea or the sense of effort decreased below 5. Work rate was adjusted each session as Borg ratings and heart rate evolved		Median duration between clinical decision and surgery was 25 days, which allowed a median of 8 high-intensity interval training sessions to be performed over 3 weeks. Adherence was 87%. Although 30% had COPD the training was well tolerated, indicating the possibility of its regular application in pulmonary rehabilitation programmes
Two week multimodal prehabilitation program improves perioperative functional capability in patients undergoing thoracoscopic lobectomy for lung cancer: A randomised controlled trial (Randomised controlled trial) [43]	• Exercise • Nutrition • Wellbeing No model or theory base reported	Exercise: Moderate intensity aerobic training, resistance exercises and respiratory training Nutrition: Nutritional counselling with whey protein supplementation Wellbeing: Mental relaxation including imagery and visualisation with relaxing music		Exercise: A doctor of physical therapy undertook all baseline assessments Nutrition: Information not provided Wellbeing: Information not provided		Exercise: Intensity based on the rate of perceived exertion (Borg scale) and target heart rate and set to achieve moderate training. Patients given an elastic resistance band to match their fitness level Nutrition: 3 day total food recall questionnaire Patients advised to change unhealthy eating habits, avoid high-calorie and high fat diets, eat more vegetables and fruits and high-quality proteins. Whey protein powder given to patients to take within one hour after exercise to achieve an intake of 1.5 g/kg/d of protein Wellbeing: A music player with relaxing music was provided				Home based		Immediately after baseline visit (2 weeks before surgery) Exercise: Aerobic exercise; 30 min 3 × week + resistance exercises 2 × week Wellbeing: Daily prior to sleeping						Exercise: Tailored to patients fitness level Nutrition: Advice given based on a 3-day total food recall questionnaire Wellbeing: Information not provided	Improvement in 6MWD, but no differences in lung function, disability, psychological assessment, length of stay, short-term recovery quality, postoperative complications, and mortality
Precision-Exercise-Prescription in patients with lung cancer undergoing surgery: Rationale and design of the PEP study trial (Randomised controlled trial protocol) [44]	• Exercise • Wellbeing The wellbeing component is based on motivational interviewing and the social cognitive theory	Intervention arm: Exercise: Consists of 5 stages: (1) Basic transfer mobility exercise (low-moderate intensity), (2) callisthenic mobility exercise (moderate-high intensity), (3) aerobic and resistance exercise (low-moderate intensity), (4) aerobic and resistance exercise (moderate intensity), (5) aerobic and resistance exercise (high intensity) Wellbeing: Motivational and problem solving telephone calls Control arm: Patients will be encouraged to increase walking both in the pre surgery and post-surgery period as part of usual clinical care, but there will be no formalised exercise programme		A physical therapist	Exercise: Instructional exercise sheets will be given to patients demonstrating exercise modes and intensity. Patients will be given access to light weights and resistance bands, an exercise diary and an activity tracker Wellbeing: Motivational interviewing techniques, identification of barriers to exercising and problem-solving solutions, goal setting and self-monitoring					Either at home, the wellness centre or a recreational centre		Pre-surgery up to 6 months post-surgery. Exercise: Stages 1 and 2: Low intensity = 30 s-1 min 2 × day. Moderate intensity = 1–13 min 2 × day. High intensity = 1.5–2 min 4 × day Stages 3, 4 and 5: Low intensity = 10 min of aerobic exercise + 5 min of resistance exercise. Moderate intensity = 20 min of aerobic exercise + 10 min of resistance exercise. High intensity = 30 min of aerobic exercise + 15 min of resistance exercise Wellbeing: Weekly						Exercise: Based on an individual’s Activity Measure for Post Acute Care (AM-PAC) outpatient basic mobility score. It can be adjusted depending on a patients level of fatigue, muscle weakness, pain and/or shortness of breath Wellbeing: Based on motivation and self-efficacy to engage in exercise	The following will be measured at baseline, on discharge, 2 months post-surgery and at 6 months post-surgery: AM-PAC mobility score, physical function (6MWD), strength, endurance and balance (short physical performance battery), patient-reported outcomes (functional assessment of cancer therapy-lung and chronic illness therapy-fatigue, Pittsburgh sleep quality index, physical activity, nutrition and wellbeing markers, subjective social status ladders, symptoms, living condition), exercise diary, length of stay, complications, healthcare costs and smoking assessment (saliva)
A comparison of the effects of medical Qigong and standard exercise therapy on symptoms and quality of life in patients with advanced cancer (Randomised cross over study) [45]	• Exercise No model or theory base reported	Medical Qigong consisted of “Walking Qigong”. A walking exercise programme involving coordinated arm movements while in a state of deep relaxation or meditation. Patients were advised to refrain from independent resistance or cardiovascular training during this period Standard exercise therapy: Consisted of cardiovascular and resistance training exercises. No details provided re: type of exercises. Patients were also advised to walk daily and to refrain from practising Qigong		A physiotherapist. Patients were also evaluated by a second physiotherapist (not involved in training and blinded to group assignment) at three time points: baseline 0–2 weeks before starting the training periods, up to 2 weeks after completing the first arm of the study and up to 2 weeks after completing the second arm	Medical Qigong and standard exercise therapy were delivered over a 6-week period. Patients stopped for a minimum of 2 weeks and then attended for a further 6 weeks to receive the other type of intervention Medical Qigong was delivered as group sessions whereas standard exercise therapy was delivered either individually or as a group Patients kept logbooks of physical activities performed at home during both 6-week training periods					At the local hospital and at home		Over a 6 week period. Medical Qigong: 12 × 45 min face to face sessions + 1 h a day at home Standard exercise therapy: Tailored to the individual + one hour of walking daily						Tailored to a patient’s individual training intensity	51 patients consented. 36 (71%) completed baseline assessment, 24 (47%) completed the first assessment after the first exercise intervention, but only 19 (37%) completed both exercise interventions and all assessments In all cases, the beneficial effects of the exercise interventions were markedly reduced during the second interval. The order in which the interventions were performed had a significant impact on the improvement in certain symptoms
Impact of physical activity on fatigue and quality of life in people with advanced lung cancer: A randomised controlled trial (Randomised controlled trial) [46]	• Exercise • Wellbeing Based on the theory of planned behaviour	Intervention arm: A physical activity and behavioural support programme incorporating aerobic physical activity, advice about resistance exercises and behavioural support sessions. Control arm: Patients received cancer-specific education materials regarding nutrition and exercise		A physical activity consultant	Exercise: One to one supervised sessions + unsupervised home physical activity sessions The behaviour support sessions use behaviour lifestyle change principles. Patients were also given a physical activity and behaviour change guidebook to use throughout the intervention					Information not provided		For 2 months Exercise: Weekly; 30–45 min Wellbeing: Weekly; 15–30 min behaviour support sessions						Exercise: Tailored to the patient’s baseline fitness, performance status and physical activity preferences Wellbeing: Information not provided	Both groups reported more baseline physical activity than anticipated and symptoms and physical function did not deteriorate greatly over time No significant differences between the two groups in terms of fatigue or QOL. Intervention adherence was good, with 69% completing all physical activity and 75% all behavioural change sessions. Attrition occurred in both groups, mainly due to disease progression
Preoperative exercise training prevents functional decline after lung resection surgery: A randomised, single-blind controlled trial (Randomised controlled trial) [47]	• Exercise No model or theory base reported	Preoperative exercise training consisted of a combination of moderate endurance and resistance training. Patients were also asked to perform breathing exercises		Physiotherapist	Endurance training: On a cycle ergometer At the beginning and end of the training, dyspnoea and leg fatigue were logged using the modified version of the Borg Scale Resistance training: Using elastic bands and body weight exercises Breathing exercises: Using a volume-oriented incentive spirometer					Hospital based		3–5 times a week depending on the surgical date						Endurance training load was determined after a symptom-limited incremental cycle test Resistance training load was determined using a 25-maximum repetition test	Out of the 40 patients who were randomised, only 22 (55%) completed at least one postoperative evaluation and were analysed. In the prehabilitation group, mean time from baseline assessment to surgery was 54.5 days with a median of 16 sessions. Patients in the prehabilitation group were able to maintain and/or increase their baseline values in all the parameters examined, whereas in the control group there was a progressive decline throughout follow-up, especially in exercise capacity
Short-term preoperative exercise therapy does not improve long-term outcome after lung cancer surgery: A randomised controlled study (Randomised controlled trial) [48]	• Exercise No model or theory base reported	HITT training program. Patients in both the intervention arm and the control group were given advice regarding active mobilisation and risk factor management (e.g., healthy nutrition and smoking and alcohol cessation)		Physiotherapist	Using a cycle ergometer					Hospital based		Pre-operatively 3 × week						The work rate was adjusted on each session according to an individual’s maximal heart rate	Short-term preoperative rehabilitation with HIIT did not improve pulmonary function and aerobic capacity measured at 1 year after lung cancer resection
Short-term Preoperative High-Intensity Interval Training in patients awaiting lung cancer surgery: A randomised controlled trial (Randomised controlled trial) [49]	• Exercise No model or theory base reported	HITT training program. Patients in both the intervention arm and the control group were given advice regarding active mobilisation and risk factor management (e.g., healthy nutrition and smoking and alcohol cessation)		Physiotherapist	Using a cycle ergometer				Hospital based in an outpatient clinic		Pre-operatively 3 × week							The work rate was adjusted on each session according to an individual’s maximal heart rate	A HIIT programme helps prepare patients before lung cancer resection by enhancing their physical fitness. However, the HIIT programme did not improve postoperative clinical outcomes
Is preoperative protein-rich nutrition effective on postoperative outcome in non-small cell lung cancer surgery? A prospective randomised study (A prospective randomised study) [50]	• Nutrition No model or theory base reported	Patients were given an immune modulating formulae (enriched with arginine, omega-3 fatty acids and nucleotides) for 10 days		Information not provided	Information not provided				Information not provided					Information not provided				Information not provided	Statistically significant difference in complication rates, mean tube drainage times and in drop in albumin levels in the intervention arm compared to the control group
Effect of prehabilitation on ventilatory efficiency in non–small cell lung cancer patients: A cohort study (Cohort study) [51]	• Exercise Not based on a model or theory, but based on research that pulmonary prehabilitation improves postoperative risk factors and ventilatory inefficiency > 35 is a high risk factor for postoperative complications	Endurance training, peripheral and inspiratory muscle strengthening		Information not provided	Endurance training: Via a cycle ergometer or a treadmill. Peripheral muscle strengthening: Information not provided. Inspiratory muscle strengthening: Using a threshold valve Patients were also taught bronchial drainage techniques and directed coughing				In an ambulatory setting					Prior to surgery. Endurance training and peripheral muscle strengthening: 90 min 3–5 × week. Inspiratory muscle strengthening: 15 min daily				Load / intensity / resistance increased according to individual tolerance	Prehabilitation did not change ventilatory efficiency. 15 or more sessions of prehabilitation seems to be a rational threshold to improve other CPET outcomes, while remaining applicable in clinical practice
Impact of prehabilitation on morbidity and mortality after pulmonary lobectomy by minimally invasive surgery: A cohort study (Cohort study) [52]	• Exercise No model or theory base reported	Endurance exercise, muscular strengthening and inspiratory muscle strengthening		Two physiotherapists	Endurance exercise and muscle strengthening was performed on a cycle ergometer Inspiratory muscle strengthening was performed using a resistive valve				Hospital based				Pre-operatively: 90 min 3–5 × week			Endurance exercise was tailored to an individual’s ventilator threshold			Although significant differences were observed in the prehabilitation group in terms of a reduction in post-operative complications and Clavien-Dindo classification, no significant differences were seen in length of stay and severity of complications between groups
Application and practice of trimodal prehabilitation model in preoperative management of patients with lung cancer undergoing video-assisted thoracoscopic surgery (Prospective study) [53]	• Exercise • Nutrition • Wellbeing No model or theory base reported	Exercise: Aerobic exercise and stair-climbing training. Deep breathing training and abdominal breathing exercise. Nutrition: Whey protein supplement drink 1 h after exercise Wellbeing: Relaxation training		Nursing staff Dietitian	Exercise: Information not provided Nutrition: Using the Nutrition Risk Screening tool. Wellbeing: Using a professional scale to evaluate psychological state and via relaxation training				Information not provided						Exercise: Aerobic exercise: 30 min 2 × day. Stair climbing: 2 × day Deep breathing training and abdominal breathing exercise: 10–15 min 3 × day. When the perceived fatigue was heavy, the exercise intensity was reduced Nutrition: Information not provided Wellbeing: 20 min daily The intervention ended after the first week of the operation			Nutritional input was tailored to the individual based on the Nutrition Risk Screening tool No information provided for exercise and wellbeing	The application of a trimodal prehabilitation model for the preoperative management of patients with lung cancer undergoing video-assisted thoracoscopic surgery is conducive to improving the functional state and psychological state, preventing complications and improving nursing satisfaction
Pre-treatment optimisation with pulmonary rehabilitation in lung cancer: Making the inoperable patients operable (Prospective observational study) [54]	• Exercise Not based on a model or theory, but on the hypothesis that older patients may have frailty and smoking related cardiopulmonary disease with reduced pulmonary function, which could impair post-operative ventilatory function predisposing them to dyspnoea, cardiopulmonary complications and death. Patients with significant dyspnoea, poor performance status or poor pulmonary function are considered in-operable	Comprised of four main elements (1) Respiratory muscle training and breathing exercises (2) Cardiovascular exercises (3) Education—Health education and smoking cessation advice (4) Pharmacology agents—Where necessary bronchodilator therapy was provided		Trained cardiothoracic physiotherapists	Information not provided				At the prehabilitation centre or outreach unit and home based						Over 2–4 weeks. 70 min face to face sessions with cardiovascular exercises 2 × week + home based respiratory muscle training and breathing exercises 3 × day			Individual training zones were calculated for the cardio-vascular exercises. Exercise intensity was based on the rate of perceived exertion (Borg scale) and target heart rate	Following optimisation with prehabilitation, 84.2% of the high-risk patients were ready to proceed with radical treatment. 42.8% of patients underwent surgery No significant differences in post-operative complications, length of hospital stay or mortality between the low and high risk groups
Neoadjuvant prehabilitation therapy for locally advanced non–small-cell lung cancer: Optimising outcomes throughout the trajectory of care (Retrospective study) [55]	• Exercise • Nutrition • Wellbeing No model or theory base reported	Exercise: Moderate-vigorous intensity aerobic training and resistance training Nutrition: Patients screened using validated tools, advised to aim for 1.5 g/kg/d of protein and prescribed whey protein supplements if required Wellbeing: Relaxation exercises based on imagery, visualisation and deep breathing to help reduce anxiety		Exercise: Certified kinesiologist Nutrition: Dietitian Wellbeing: Psychology trained personnel	Exercise: According to a patient’s preferred type of aerobic training Nutrition: All patients screened using the PG-SGA and the Nutritional Risk Screening tool. Daily protein calculated at 1.5 g/kg ideal body weight Wellbeing: A compact disc with relaxation exercises provided				Home-based unsupervised programme						Immediately after baseline assessment (approximately four weeks prior to surgery) and up to eight weeks after surgery. Exercise: 30 min 3 × week + resistance training 3 × week Nutrition: 1.5 g/kg ideal body weight of protein per day + protein supplements within 1 h of exercise Wellbeing: 2–3 × week			Exercise: Tailored to a patient’s preferred type of exercise and fitness level Nutrition: Advice given based on a 3 day food diary completed at the time of enrolment Wellbeing: Information not provided	The prehabilitation programme was individualised and home-based, which allowed patients to complete the programme at home at any convenient time, simplifying the already demanding schedule of a patient undergoing neoadjuvant therapy. Only 1 patient dropped out after initiation of the prehabilitation programme, demonstrating good feasibility
Malnourished lung cancer patients have poor baseline functional capacity but show greatest improvements with multimodal prehabilitation (Retrospective study) [56]	• Exercise • Nutrition • Wellbeing No model or theory base reported	Exercise: The home-based training included moderate intensity aerobic training, resistance exercises and flexibility exercises Nutrition: Individualised plan to meet each patients nutritional needs + whey protein supplementation to achieve a total protein intake of 1.2–1.5 g/kg/d Wellbeing: Patients given techniques aimed at reducing anxiety, such as relaxation exercises based on imagery, visualisation and deep-breathing exercises		Exercise: Kinesiologist Nutrition: Dietitian Wellbeing: Psychology trained personnel	Exercise: Patients provided with an information booklet with instructions and figures on all elements of the program and exercise progressions. The booklet also included a journal to record all activities related to the program. Nutrition: A comprehensive dietary assessment based on a 3-day food diary, anthropometry, nutrition impact symptoms, biochemistry and a nutrition focused physical exam. Instructions included eating well-balanced meals with a focus on protein intake. Wellbeing: One to one sessions and a compact disc with relaxation exercises for home				Hospital based combined with unsupervised exercise sessions at home						4 weeks prior to surgery. Exercise: 30 min 5 × week Nutrition: Information not provided Wellbeing: 2–3 × week			Exercise: Individualised based upon initial assessments Nutrition: Individualised dietary advice Wellbeing: Tailored according to a patients needs	Patients classified with moderate or high nutrition risk (according to the PG-SGA) exhibited significantly lower physical performance at baseline, including functional capacity, timed get up and go, grip strength and self-reported physical activity levels compared with low-nutrition-risk patients. High nutrition risk patients have the most to gain (functionally) from multimodal prehabilitation compared with low nutrition risk patients
Feasibility and outcomes of a real-world regional lung cancer prehabilitation programme in the UK (Feasibility study) [57]	• Exercise • Nutrition • Wellbeing Based on the principles of NHS England’s Personalised Care model	Exercise: All patients triaged into ‘universal’ or ‘targeted’ pathways. For the universal pathway, patients could exercise independently with weekly monitoring with an exercise specialist. Exercise prescriptions included high-intensity interval training and resistance training Nutrition and wellbeing: Assessed at baseline and at intervals throughout the programme. A three-tier risk assessment (low, medium and high) was used to identify those in need of nutritional or wellbeing support and each category received simple interventions or onward referral when required		Exercise specialists (level four cancer rehabilitation qualified exercise practitioners) were responsible for screening patients and delivering all components of the intervention	Patients initially contacted by telephone to organise a face-to-face appointment at a first assessment clinic. Baseline assessments took place face-to-face and an individualised programme was prescribed				Patients could complete their individualised programme at any one of the 87 local leisure centres						Exercise prescriptions for the targeted pathway included 3 supervised group gym sessions x No information is provided on the length of the prehabilitation period. Baseline functional and QOL assessments were repeated immediately before the date of surgery After treatment, a 12 week postoperative rehabilitation programme was provided			Individualised prehabilitation programme prescription for all tri modal components	Of the 377 patients referred, overall participation on an intention to treat basis was 47.7%. Median interval from assessment to surgery was 36 days. There were no adverse events. Statistically significant improvements in the Incremental Shuttle Walk Test (ISWT), 6MWT, 60 min sit to stand test (STS), Hand Grip Dynamometry (HGD), World Health Organisation Disability Assessment Scheduled (WHODAS), Self-Efficacy for Exercise (SEE), International Physical Activity Questionnaire (IPAQ), and the European Quality of Life Five Dimensions (EQ-5D) scores
Feasibility of setting up a pre-operative optimisation ‘prehab’ service for lung cancer surgery in the UK (Feasibility study) [58]	• Exercise Not based on a model or theory, but based on the hypothesis that respiratory function could be improved by optimising treatment for comorbidities, potentially tipping the balance between a patient being deemed not fit enough for surgery and being considered a surgical candidate	The prehabilitation programme was based around the mainstays of COPD management: optimising inhaled therapy, smoking cessation and pulmonary rehabilitation (including progressive muscle resistance and aerobic training)		Oncology outpatient physiotherapy team, local community respiratory team or cardio-respiratory physiotherapy team depending on patient choice, need and availability	Information not provided				One-to-one or group sessions depending on patient choice, need and availability						Referral was made as early as possible. For the majority of patients, the duration was driven by the pragmatic constraints of the 62-day lung cancer pathway. The pragmatic approach around location, nature and duration of programme led to significant variation in the nature of individual patients programmes			Tailored according to patient choice, need and availability	Median number of sessions for 35 patients seen purely by the outpatient oncology physiotherapy team was three over a median of 22 days. Eight patients (16%) underwent inpatient prehabilitation, with a median duration of eight days. This variance was predominantly driven by the scheduled surgical date. 13 patients (26%) were unnecessary or inappropriate referrals Improvement in surgical rates from 12.8% at the inception of the study to 29.8% at the end
A feasibility study of an unsupervised, pre-operative exercise program for adults with lung cancer (Feasibility study) [59]	• Exercise No model or theory base reported	Patients received a verbal and written exercise prescription from their surgeon: “Do any moderately-intense aerobic physical activity (e.g., walking, jogging, stair climbing, upper body ergometer, stationary bicycle) for 30 min a day and for 5 days each week. While doing the activity, you should be working hard enough that it is difficult to speak more than a few words at a time. You may need to start slowly (e.g., 5–10 min at a time), but as you get stronger you can increase your activity so that you exercise for 30 min at a time.”		Surgeon and a project co-ordinator	Each patient was given a Garmin Vivoactive heart rate device. Patients were assigned an email address and password which was used as a login for the Garmin Connect Mobile App. The project coordinator downloaded the application onto patient phones and activated the fitness device during enrolment Patients were asked to synchronise and charge the device daily and wear the device at all other times including showering and sleeping Patients recorded their aerobic exercise sessions within a paper-based log				Home based						Pre-operatively 30 min 5 × week			Patients were allowed to choose the type of activity that they found most feasible or enjoyable	81% recruitment rate with 79% completing the pre-operative assessments. Only 29% completed the exercise log and 14% experience adverse events related to the device 79% reported at least one aspect of the fitness device that they disliked and 29% reported at least one thing they did not understand regarding the device
Pre-radiotherapy daily exercise training in non-small cell lung cancer: A feasibility study (Feasibility study) [60]	• Exercise No model or theory base reported	Structured exercise training immediately prior to radiotherapy. This consisted of three exercise phases. Phase one and three: interval training. Phase two: continuous cycling		An exercise physiologist or physiotherapist	Supervised, individualised exercise using an ergometer cycle. All patients wore heart rate monitors during the exercise sessions and were given Garmin® vívosmart® heart rate activity trackers to use during the course of radiotherapy				Next to the accelerator when a patient attended for radiotherapy treatment						The intervention period was equal to the patient’s number of radiotherapy sessions over a seven week period, with each session lasting 20 min			The exercise sessions were tailored to the patient’s fitness level	The intervention comprised of 31 prescribed exercise sessions over the seven-week period. Overall attendance rate to exercise was 90%. Of the 90% attendance, the adherence rate to full exercise participation was 88.1% and was performed by a modified program due to early exhaustion, pause during the exercise session or practical reasons (e.g. earlier start of radiotherapy on a given day) 2 patients were hospitalised due to chemotherapy adverse events. No adverse events were observed during the exercise sessions
Patients’ and healthcare professionals’ views on a pre-and post-operative rehabilitation programme (SOLACE) for lung cancer: A qualitative study (Qualitative study) [61]	• Exercise • Nutrition • Wellbeing Not based on a model or theory, but based on a previous pre and post-surgical intervention study in pulmonary rehabilitation which demonstrated a reduction in post-operative complications and hospital readmissions	No specific information provided. Services provided in the SOLACE programme include patient education / written information on recovery from lung cancer surgery, smoking cessation, nutritional advice, information on thoracic surgical pathways, psychological support, pain management, financial help, links to other NHS, Macmillan and external support agencies, pre-and post-operative rehabilitation exercise classes, local exercise referrals and rehabilitation DVDs		A Macmillan lung cancer survivorship Advanced Nurse Practitioner (ANP) and a Macmillan lung cancer survivorship Advanced Therapist Practitioner (ATP)	Through the provision of personalised support depending on a patient’s support requirements, Interventions informed by the Macmillan guidance				Hospital and/or community based						Information not provided			Provision of personalised support	Pre and post-operative rehabilitation services can help improve patients’ perceived physical and psychological health and build self-confidence in their ability to self-manage There is a need to consider ways to enable prolonged access to rehabilitation services for lung cancer patients entering the follow-up stages of their care
Attitudes and perceptions to prehabilitation in lung cancer (Online cross-sectional survey) [62]	• Exercise No model or theory base reported	24 item survey sent to 198 fellows of the Royal Australasian College of Cardiothoracic Surgeons		Information not provided	Via email, college newsletters, digital press and in printed format				Throughout Australia and New Zealand						Information not provided			Information not provided	> 90% of cardio-thoracic surgeons surveyed would delay surgery for prehabilitation in order to optimise ‘high risk’ patients (those with multiple comorbidities, advanced age, positive smoking status / respiratory disease, and/or obesity) preoperatively, particularly in patients with borderline fitness for surgery. The main barriers to prehabilitation reported were patient comorbidities and access to allied health professionals
Potential effectiveness of a surgeon-delivered exercise prescription and an activity tracker on pre-operative exercise adherence and aerobic capacity of lung cancer patients (Proof of concept study) [63]	• Exercise Not based on a model or theory, but based on the hypothesis that pre-operative exercise can improve functional outcomes for lung cancer patients, but barriers associated with cost, resources, and burden make it challenging to deliver pre-operative exercise programs	Patients received a verbal and written exercise prescription from their surgeon: “Do any moderately-intense aerobic physical activity (e.g., walking, jogging, stair climbing, upper body ergometer, stationary bicycle) for 30 min a day and for 5 days each week. While doing the activity, you should be working hard enough that it is difficult to speak more than a few words at a time. You may need to start slowly (e.g., 5–10 min at a time), but as you get stronger you can increase your activity so that you exercise for 30 min at a time.”		Surgeon and a project co-ordinator	The project co-ordinator provided a written copy of the prescription on enrolment. Each patient was given a Garmin Vivoactive heart rate device. Patients were assigned an email address and password which was used as a login for the Garmin Connect Mobile App. The project coordinator downloaded the application onto patient phones and activated the fitness device during enrolment. Patients were asked to synchronise and charge the device daily and wear the device at all other times including showering and sleeping				Home based						Pre-operatively 30 min 5 × week			Patients were allowed to choose the type of activity that they found most feasible or enjoyable	Proof of concept was achieved as nearly half of the study sample achieved the minimal clinically meaningful improvement in aerobic capacity prior to surgery as a result of participation in the intervention. However, approximately 50% did not achieve this and the majority of patients fell short of achieving the prescribed weekly MVPA goal. Future work should involve the use of a triage system to identify patients who can successfully adhere to the pre-operative exercise prescription with only “low-touch” support and those who could benefit from additional resources and high-touch forms of support
Prehabilitation in thoracic cancer surgery: From research to standard of care (Quality Improvement Project) [64]	• Exercise • Nutrition • Wellbeing No model or theory base reported	The prehabilitation program included 3 steps: (1) Screening, (2) Assessment and (3) Intervention. Screening identified high-risk patients with at least one functional, nutritional, or psychological impairment. Assessment quantified the severity of the impairment to help tailor the intervention. The intervention was tailored based on specific physical, nutritional, or psychological impairments identified during the assessment phase. High-risk patients with a mild impairment received a low-intensity prehabilitation program, whereas high-risk patients with severe impairments were given a high-intensity prehabilitation program		Exercise: Kinesiologist Nutrition: Dietitian Wellbeing: Nurse specialist with specific training in psycho-social support for patients with cancer	Exercise: Screening—6MWT and the Duke Activity Status Index (DASI). Assessment—CPET Nutrition: Screening—Involuntary weight loss > 10% in 6 months and/or reduced dietary intake < 50% over the previous weeks and/or nutrition related symptoms (poor appetite, dysphagia, vomiting or and constipation over the last weeks) and/or low handgrip strength: < 20th percentile of normative value. Assessment: PG-SGA Wellbeing: HADS questionnaire				Hospital based						Information not provided			All interventions were tailored to each individual	58 patients showed at least one or more physical, nutritional, or mental impairment and progressed onto the assessment phase of the program. 23 patients were categorised as low risk. 45 high-risk patients received a one-month personalised prehabilitation program. 16 of these received a trimodal program and 22 received a nutrition and exercise program The median duration of prehabilitation was 30 days

### Overview of the studies

The 31 studies included in this review comprised of fourteen randomised controlled trials [34–36, 38, 40–43, 45–50], four feasibility studies [57–60], three registered protocols [37, 39, 44], two cohort studies [51, 52], two prospective studies [53, 54], two retrospective studies [55, 56], one qualitative study [61], one cross sectional survey [62], one proof of concept study [63] and one quality improvement study [64].

The largest number of studies originated from Canada (*n* = 6). The origins of the other studies included France (*n* = 4), United Kingdom (*n* = 4), China (*n* = 3), Switzerland (*n* = 3), United States of America (*n* = 3), Australia (*n* = 2), Denmark (*n* = 2), Spain (*n* = 2), Ireland (*n* = 1) and Turkey (*n* = 1).

Sample sizes ranged from 15 to 377 lung cancer patients with a mean age range of 46 – 72 years of age.

### How does the literature within the field of lung cancer describe the structure of prehabilitation?

All studies except one [50] included in this review featured an exercise component as part of a prehabilitation programme. Sixteen studies were uni-modal, focusing solely on exercise prehabilitation [38, 40–42, 45, 47–49, 52, 54, 58–60, 62, 63]. Ten out of the 31 studies described multimodal prehabilitation interventions using a tri-modal approach, incorporating nutrition, exercise and psychological wellbeing [35, 36, 39, 43, 53, 55–57, 61, 64]. Other studies incorporated an exercise and psychological component (*n* = 3) [34, 44, 46] or an exercise and nutrition component (*n* = 1) [37]. A single study focused solely on nutrition prehabilitation [50], but there were no uni-modal psychological wellbeing prehabilitation intervention studies. See Table 3.

All prehabilitation interventions varied in terms of programme setting, type of intervention, patient criteria, intervention delivery, duration of prehabilitation and measured outcomes. Nine out of the 31 studies provided a comprehensive description of all aspects of the prehabilitation programme [36, 40, 44, 45, 47, 52, 59, 60, 63]. In relation to programme setting, most interventions were delivered in a hospital setting (*n* = 9) [37, 47–49, 51, 52, 58, 60, 64] or provided flexibility between a hospital setting and remote supervision (*n* = 9) [36, 39, 40, 44, 45, 54, 56, 61, 62]. Six of the 31 studies used remote supervision [35, 43, 55, 57, 59, 63]. In the remaining studies, the programme setting was unclear (*n* = 7) [34, 38, 41, 42, 46, 50, 53].

### Exercise

All prehabilitation programmes that included an exercise intervention used a baseline physical fitness assessment to develop an individualised exercise prescription. The type of baseline physical fitness assessment used was variable throughout all of the studies, ranging from cardio-pulmonary exercise testing to more general elements such as muscle strength and activity questionnaires. More than 50% of the studies described the exercise intervention as a combination of both aerobic and resistance exercise to improve cardiorespiratory fitness and muscle strength. Other exercise interventions included High Intensity Interval Training (HIIT) and medical Qigong. Breathing exercises such as Respiratory Muscle Endurance Training (RMET) were also described under the term exercise, alongside aerobic exercise interventions.

### Nutrition

Tailored dietetic advice was provided in all prehabilitation programmes which included a nutritional component (*n* = 12) [35–37, 39, 43, 50, 53, 55–57, 61, 64]. In eight studies, this was based on an individualised assessment, which was undertaken by a registered dietitian [35–37, 39, 53, 55, 56, 64]. Only three studies [35, 55, 64] stated using validated tools such as the Patient Generated Subjective Global Assessment (PG-SGA). However, the use of nutritional screening tools to assess malnutrition risk was not frequently described. The majority of studies (*n* = 8) [35–37, 39, 43, 53, 55, 56] focused on increasing dietary protein, often recommending the use of protein supplements. Two interventions focused on the use of a fish oil supplement [36, 50].

### Psychological wellbeing

Only three studies described the validated tools that were used to establish baseline psychological wellbeing. These included the Hospital Anxiety and Depression Score (HADS) [53, 55] and the Short Form (SF) 36 questionnaire [47]. There was large variation in the description of the psychological interventions used. Some studies used the terms ‘support’ and ‘coping strategies’, whereas in other studies, more specific techniques such as relaxation, imagery, visualisation, cognitive behavioural therapy and motivational interviewing were described.

### How does the literature within the field of lung cancer describe the personalisation of prehabilitation interventions?

A number of studies described tailoring the exercise, nutrition and/or psychological wellbeing prehabilitation intervention to an individual (*n* = 28), but there was large variation across all studies in how this was fully conceptualised and achieved. The personalisation of the prehabilitation intervention mostly referred to a starting point variation along a continuum, e.g. intensity of exercise in relation to baseline fitness. This was typically considered at a single point, usually at baseline and not reviewed.

Only two studies [44, 46] in this review reported using theory to underpin the design of this complex intervention. The two theories that were described were the theory of planned behaviour and social cognitive theory, respectively.

### What are the actual outcomes for lung cancer patients participating in a prehabilitation programme?

Twenty-six out of the 31 studies included in this review focused on lung cancer surgery. The remaining studies included chemotherapy (*n* = 2) [38, 45], radiotherapy (*n* = 1) [60], neoadjuvant treatment (*n* = 1) [55] or had a specific focus on quality of life (*n* = 1) [46] for those with advanced lung cancer.

There was a wide variety of outcomes reported amongst all interventional studies. The majority of reported actual outcomes were positive. These included improvements in functional capacity (*n* = 16) [34, 40–43, 45, 47, 49, 51, 53, 55–58, 63, 64], high adherence to the intervention (*n* = 5) [36, 42, 57, 59, 60], improvements in psychological wellbeing (*n* = 3) [38, 53, 55], reductions in post-operative complications (*n* = 5) [40, 41, 50, 52, 53], improvements in muscle strength (*n* = 3) [38, 45, 47], no adverse events (*n* = 5) [34, 57, 58, 60, 64], qualitative outcomes including increased perceived physical and psychological health benefits (*n* = 2) [61, 62], reductions in length of hospital stay (*n* = 2) [41, 64] and improvements in quality of life (*n* = 1) [58]. Conversely, five studies reported no improvement in functional capacity [35, 36, 38, 46, 48]. Furthermore, some studies described no improvement in quality of life (*n* = 4) [36, 41, 45, 46], length of hospital stay (*n* = 5) [35, 43, 51, 52, 54], post-operative complications (*n* = 5) [43, 49, 51, 54, 64], survival (*n* = 3) [43, 46, 48] or psychological wellbeing (*n* = 2) [43, 45]. Only one study [59] reported an unintended outcome of their intervention which was related to an adverse reaction to the device used to monitor heart rate.

The intended outcomes described within the three protocol papers included in this review [37, 39, 44] are similar to the actual outcomes reported from the interventional studies as shown above. These include improvements in functional capacity (*n* = 2) [37, 44], improvements in quality of life (*n* = 2) [37, 44], reductions in hospital length of stay (*n* = 2) [39, 44], reductions in post-operative complications (*n* = 3) [37, 39, 44] and qualitative outcomes including self-efficacy (*n* = 1) [44]. All three randomised controlled trials for which protocols have been published [37, 39, 44] include health economics in their planned outcomes. These include treatment related costs as well as costs from a healthcare professional perspective. This will provide valuable information on the economic implication of the adoption of prehabilitation programmes for patients with lung cancer.

Specific to the oncological lung cancer treatment pathway, a multi-modal prehabilitation programme delivered remotely during neoadjuvant treatment led to a significant improvement in functional capacity and psychological wellbeing [55]. In comparison, a uni-modal exercise prehabilitation intervention for 110 lung cancer patients with advanced inoperable lung cancer undergoing chemotherapy showed no significant difference in functional capacity. However, there were significant improvements in strength and psychological wellbeing [38]. Nevertheless, these results should be interpreted with caution owing to high attrition, poor adherence and anticipated attenuation of decline amongst this cohort.

## Discussion

To our knowledge, this is the first scoping review that aims to summarise the evidence on prehabilitation in the lung cancer pathway using a realist approach.

Our review provides a summary of several interventional studies and three ongoing randomised controlled trials for which protocols have been published.

The majority of studies in this review had a surgical focus and demonstrated that prehabilitation before lung cancer surgery is feasible and is associated with physiological and psychological benefits. Our findings reveal that there are only a few studies involving lung cancer patients undergoing oncological treatment, despite 70–80% of people with lung cancer receiving non-surgical treatment within the UK [65].

Our analysis of the contexts, mechanisms and outcomes for prehabilitation provide useful insights into the factors that need to be considered in the design and implementation of prehabilitation for patients with lung cancer.

Prehabilitation is a complex intervention. It is widely understood that the success of a complex intervention depends on the theory underpinning its design [19], which helps to explain the mechanisms underlying an individual’s behaviour, based on what works for them and their circumstances [22]. However, only two studies in this review described using theory to underpin the design of this complex intervention [44, 46]. Similarly, there are only two completed studies which used a qualitative approach [61, 62], with only one evaluating the acceptability of their interventions, despite this being an important consideration for complex interventions [61, 66]. Although the qualitative literature in this field sheds some light on some of the factors which might influence engagement with prehabilitation, it does not fully illustrate the complexity of delivering prehabilitation.

Using a realist lens, this review has identified the importance of a personalised approach to prehabilitation. Whilst the personalisation of the prehabilitation intervention was often stated by the studies reviewed, and generally viewed as a positive factor, how it was fully conceptualised and achieved was less clear. The personalisation of the intervention mostly referred to a starting point variation along a continuum, e.g. intensity of exercise in relation to baseline fitness. No studies considered personalisation to patient-led values, needs, goals, support structures and beliefs. This is a considerable gap, given that initiation and adherence to any intervention is determined by behavioural, psychological, physiological, environmental and social factors [20–23], especially when research findings with well-intentioned patients need to be translated to clinical practice with the full range of real-world complexities and comorbidities [23].

Personalisation was mostly considered at a single point, typically at baseline. This is typical in pre-surgical prehabilitation, where it is a relatively brief and linear process from baseline to a defined, one-off target (surgery). However, prehabilitation during oncological treatment is a prolonged and undulating 'marathon', during which the patients other roles, values and needs have to be considered with learning and adaptation along the journey. An adaptable model and practice of ongoing, collaborative personalisation therefore needs to be explicitly defined and implemented. To address this, future research could utilise the Adversity, Restoration and Compatibility (ARC) framework [21] to help underpin a personalised and collaborative prehabilitation programme. The ARC framework provides a synthesised view of how people conceptualise the personal experience of living with and beyond cancer, namely as an ongoing process of learning about their evolving challenge (Adversity), learning how to cope effectively (Restoration) and adapting one's identity (Compatibility), in parallel. These broad themes, derived from qualitative synthesis of over 70 primary studies of patient narratives, are consistent with psychological adjustment theory [67] and may provide a structure for personalising prehabilitation that is patient-centred rather than a purely logistical or psychometric approach. This could be key to empower patients to maintain progress through the longer, more variable context of peri-oncological prehabilitation. This theory-led approach is further supported by Faithfull et al., 2019 [5] and The Medical Research Council (MRC) framework for developing and evaluating complex interventions [68] which suggests that future studies should use a conceptual framework to help guide intervention design and thereby maximise outcomes.

We acknowledge the limitations of this review. Firstly, we recognise that some studies may have been missed by database searching or were published after the search date. Secondly, the extrapolation of findings is limited owing to context dependency e.g. it may be difficult to extrapolate the results from a pre-surgical setting to those with a poor prognosis. Thirdly, only a few studies included in this scoping review provided a comprehensive description of all aspects of the prehabilitation programme, therefore the descriptions of the interventions are limited. To counterbalance this, a realist framework of context, mechanism and outcome has been used for reporting. Furthermore, our analysis of the mechanisms and outcomes for prehabilitation provide insight into the role of prehabilitation within the lung cancer pathway.

The coronavirus pandemic has accelerated the remote delivery of prehabilitation interventions. Completed studies suggest that home-based multimodal prehabilitation is feasible and leads to improvements in a range of outcomes [43, 55, 57, 59, 63]. However, there is limited qualitative data in this field to determine whether remote delivery of prehabilitation interventions is more or less favourable than face to face or a hybrid approach.

There is potential for digital interventions within this field. Two completed studies in this review used an app for the delivery of their prehabilitation interventions [59, 63], with one study demonstrating high recruitment and attrition rates [59] and the other showing a minimally clinically meaningful improvement in aerobic capacity prior to surgery as a result of participation in the intervention [63]. However, it is important to note that in the latter study, approximately 50% did not achieve an improvement in aerobic capacity with the majority of patients falling short of achieving the prescribed weekly exercise target. Technology access, skillset and confidence strongly need to be considered prior to implementation of delivery of prehabilitation interventions [69]. Furthermore, the cost-effectiveness of a technology supported multimodal prehabilitation programme needs to be evaluated. The study by Barberan-Garcia et al. [39] aims to address this, as detailed in their published study protocol.

Whilst there will be emerging evidence from ongoing randomised controlled trials, the heterogeneity in study designs, programme setting, type of intervention, patient criteria, intervention delivery, duration of prehabilitation and measured outcomes is significant. There are no trials which have the same set of primary and secondary outcomes. The lack of standardisation across interventions and outcome measures makes it difficult to conclude benefit across the whole lung cancer pathway. The inability to draw significant improvement benefit of prehabilitation due to the heterogeneity of studies has also been seen in systematic reviews in breast cancer [70], pancreatic cancer [71] and hepatobiliary cancers [72].

## Conclusion

This scoping review demonstrates that there is evidence for providing prehabilitation for patients with lung cancer, particularly in the surgical domain. However, there is a lack of clinical trials which provide definitive evidence on the programme design, setting, type of intervention, patient criteria, delivery and duration. This therefore makes it difficult to conclude significant improvement benefit.

The design and implementation of future lung cancer prehabilitation programmes should take into account factors such as patient led values, needs, goals, support structures and beliefs which can affect the delivery and engagement of interventions. The findings of this review provide important insights into these issues.

Furthermore, future research should consider the use of a conceptual framework such as ARC [21] to conceptualise the living with and beyond cancer experience to help shape and inform personalised prehabilitation services. This will enable personalised care to be given from the outset and help support identification of the ideal prehabilitation model and delivery options to optimise both health and economic outcomes. This will enable patient empowerment and engagement towards self-managed behaviours and thus, optimise long-term health.

## Data Availability

All data generated or analysed during this study are included in this published article.
